# Supplementary material for: Robust tissue pattern formation by coupling morphogen signal and cell adhesion
Source: EMBO Rep. 2024 Sep 27;25(11):4803–26. doi: 10.1038/s44319-024-00261-z (PMC11549100; doi:10.1038/s44319-024-00261-z)
Supplement: Supplementary file 1 — Appendix [file 44319_2024_261_MOESM1_ESM.pdf]

Appendix for

Robust tissue pattern formation by coupling morphogen signal and cell adhesion

Kosuke Mizuno *et al.*

Table of Contents

Appendix Fig. S1. Ectopically activated cells in imC and imC^{Ecad} cells. 2
Appendix Fig. S2. Estimation of synthetic morphogen concentration. 3
Appendix Fig. S3. Synthetic tissue domain formation by morphogen-induced cadherin. 4
Appendix Fig. S4. Tunable tissue domain formation by morphogen-induced cadherin. 6
Appendix Fig. S5. Simulation analysis of the mathematical model. 7

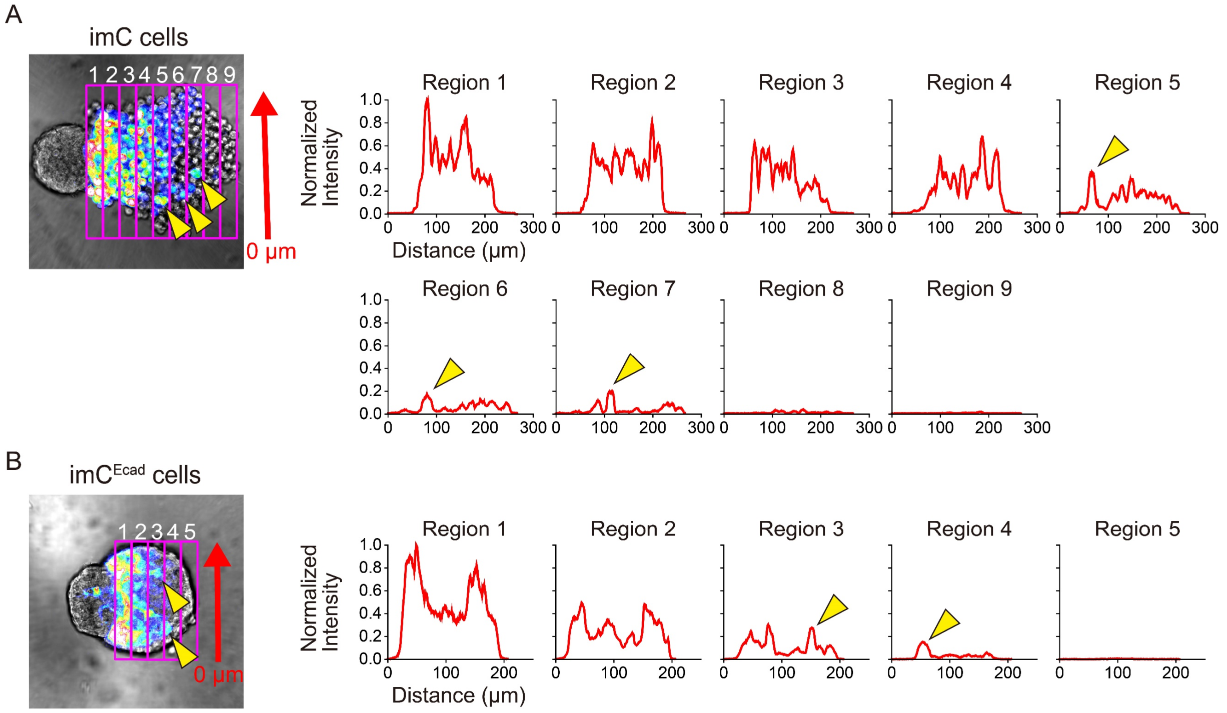

Appendix Fig. S1. Ectopically activated cells in imC and imC^{Ecad} cells.

(A) Establishment of imC cells: imC cells express the GFP anchor protein, which captures GFP on the cell surface, and the anti-GFP synNotch receptor, which induces the mCherry reporter upon recognizing the captured GFP. The GFP-anchor protein and anti-GFP synNotch receptor utilize different nanobodies (LaG2 and LaG17, respectively) that recognize different epitopes on GFP. To confirm the induction of mCherry reporter by the anti-GFP synNotch receptor, imC cells were stimulated with K562 cells expressing membrane-tethered GFP (K562^{mGFP}) (*18*) and analyzed using flow cytometry. **(B)** Quantification of EACs in the imC spheroids generated at distant positions from the GFP-secretor spheroid. Nine rectangular ROIs with a width of 36 µm were set vertically in the direction of GFP diffusion. The mCherry distributions in each ROI were quantified from bottom to top along the red arrows. The synthetic gradients are expected to spread isotropically, but abnormally activated EACs appear in regions 5–7, as indicated by the yellow arrowheads. **(C)** Quantification of EACs in imC^{Ecad} spheroids. Five rectangular ROIs were set to quantify mCherry distribution vertically in the direction of GFP diffusion. imC^{Ecad} showed a trend in which cells at the edges were more likely to be activated than those at the center. Some EACs appear in regions 3–4, as indicated by the yellow arrowheads.

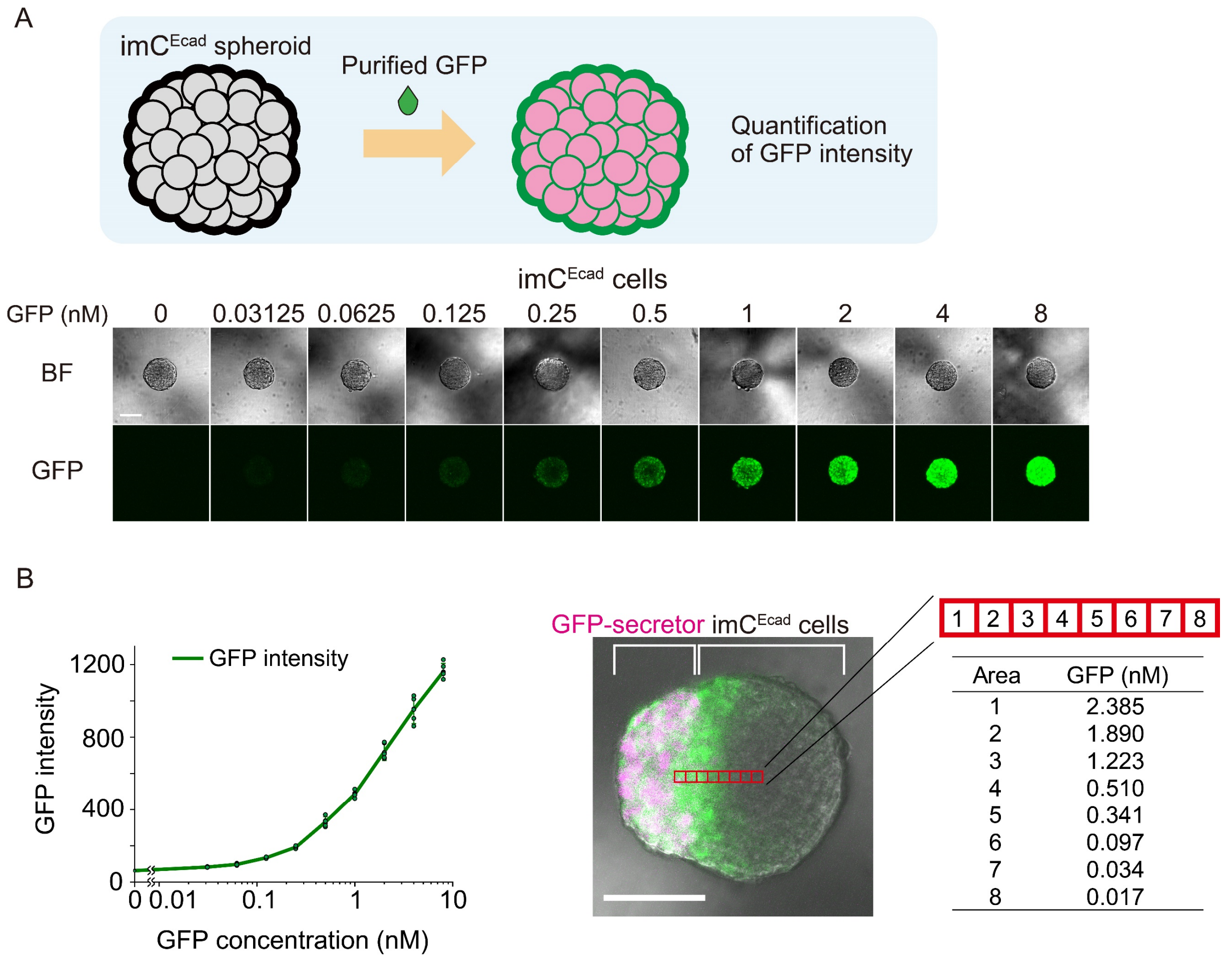

Appendix Fig. S2. Estimation of synthetic morphogen concentration.

(A) Quantification of GFP intensity in the imC^{Ecad} spheroids in the presence of various concentrations of GFP. These images are from the same samples as in Fig. EV3B. While Fig. EV3B shows bright-field and mCherry channels, this figure presents bright-field and GFP channels. Spheroids containing 100 imC^{Ecad} cells were cultured in media containing 0–8 nM GFP. Bright-field and GFP channels of the imC^{Ecad} spheroids. Scale bar: 100 µm. **(B)** Estimation of GFP gradient concentration. Based on the above results, a calibration curve was plotted to establish the relationship between GFP intensity in the spheroids and the concentration of purified GFP added to the medium. Concentrations of GFP were assessed by measuring the GFP intensity in the square ROI (1–8) of the imC^{Ecad} spheroids and estimated using the calibration curve. GFP-secreting cells are labeled with a magenta pseudocolor. Scale bar: 100 µm. The table provides the estimated GFP concentrations in the square ROI. Experiments were performed with 4-6 replicates, and data are presented as mean ± SD.

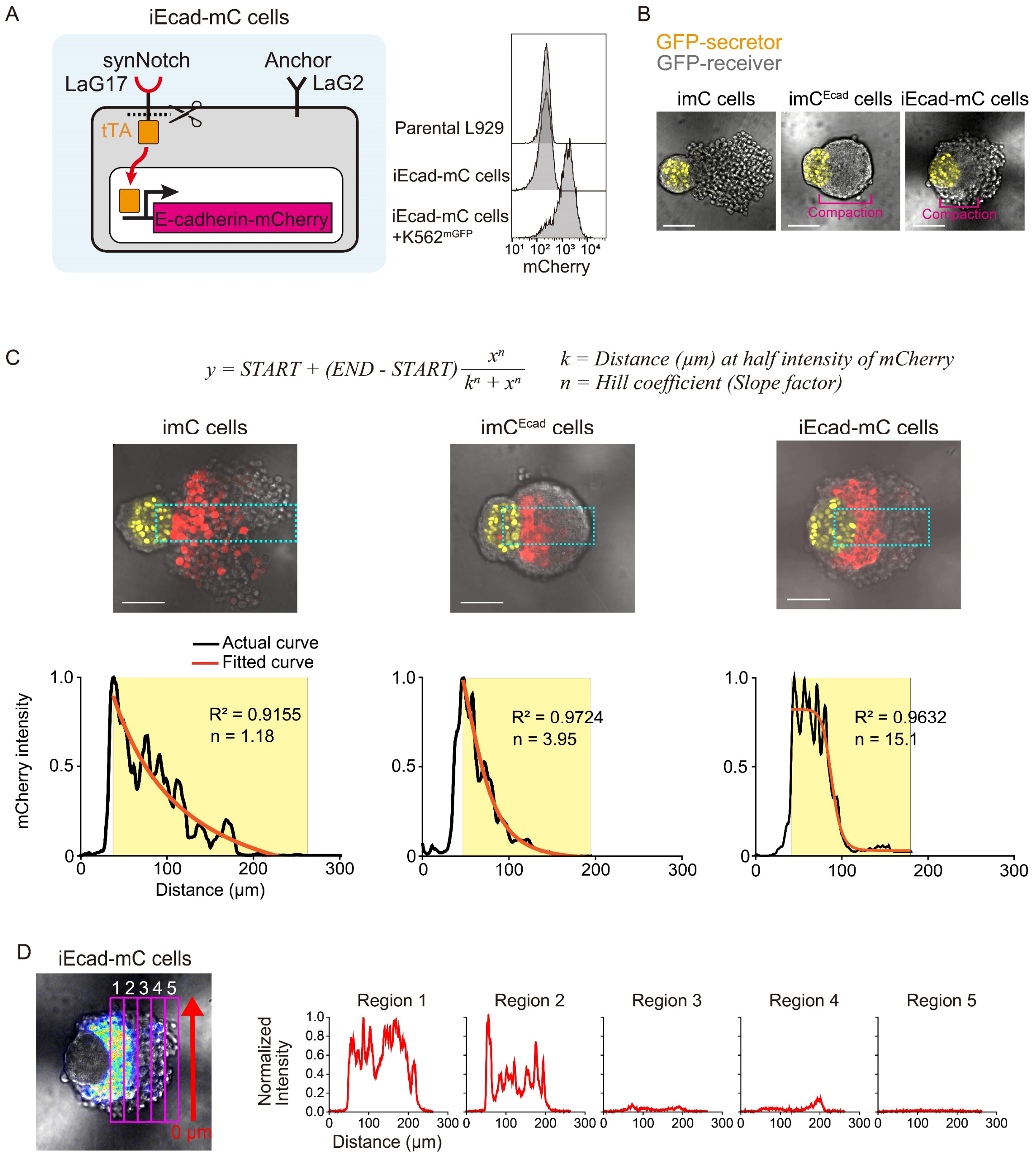

Appendix Fig. S3. Synthetic tissue domain formation by morphogen-induced cadherin.

(A) Establishment of the iEcad-mC cells: iEcad-mC cells express the GFP-anchor protein and anti-GFP synNotch receptor, which recognizes the captured GFP to induce E-cadherin-mCherry. iEcad-mC cells were stimulated with $K562^{mGFP}$ to confirm the induction of E-cadherin expression by the anti-GFP synNotch receptor using flow cytometry. **(B)** Compaction of synthetic tissue domain. Images of imC, imC^{Ecad}, and iEcad-mC spheroids co-cultured with GFP secretor spheroids are shown. Each snapshot depicts a bright-field image of Fig. 1C, 2C, or 3B at 48 h with GFP-secreting

cells indicated in yellow pseudocolor. Compaction areas are highlighted. Scale bar: 100 μm. **(C)** Quantification of spatial distribution of the mCherry reporter using the Hill coefficient. The mCherry profiles in the rectangular ROI of each spheroid (imC, imC^{Ecad}, and iEcad-mC cells) were fitted with the Hill function built into OriginPro software. The Hill coefficients of the fitted lines (orange) were calculated and plotted. Both imC and imC^{Ecad} spheroids showed a graded distribution of mCherry, resulting in low Hill coefficient values. In contrast, iEcad-mC spheroids showed a switch-like transition of mCherry, leading to high Hill coefficient values, indicating that activated and inactive domains were formed with sharp boundaries. Scale bar: 100 μm. **(D)** Quantification of EACs in the iEcad-mC spheroids (similar approach as Appendix Fig. S1A-B). To quantify mCherry distribution five rectangular ROIs were set vertically in the direction of GFP diffusion. No EACs were detected outside the tissue boundary in regions 3–5.

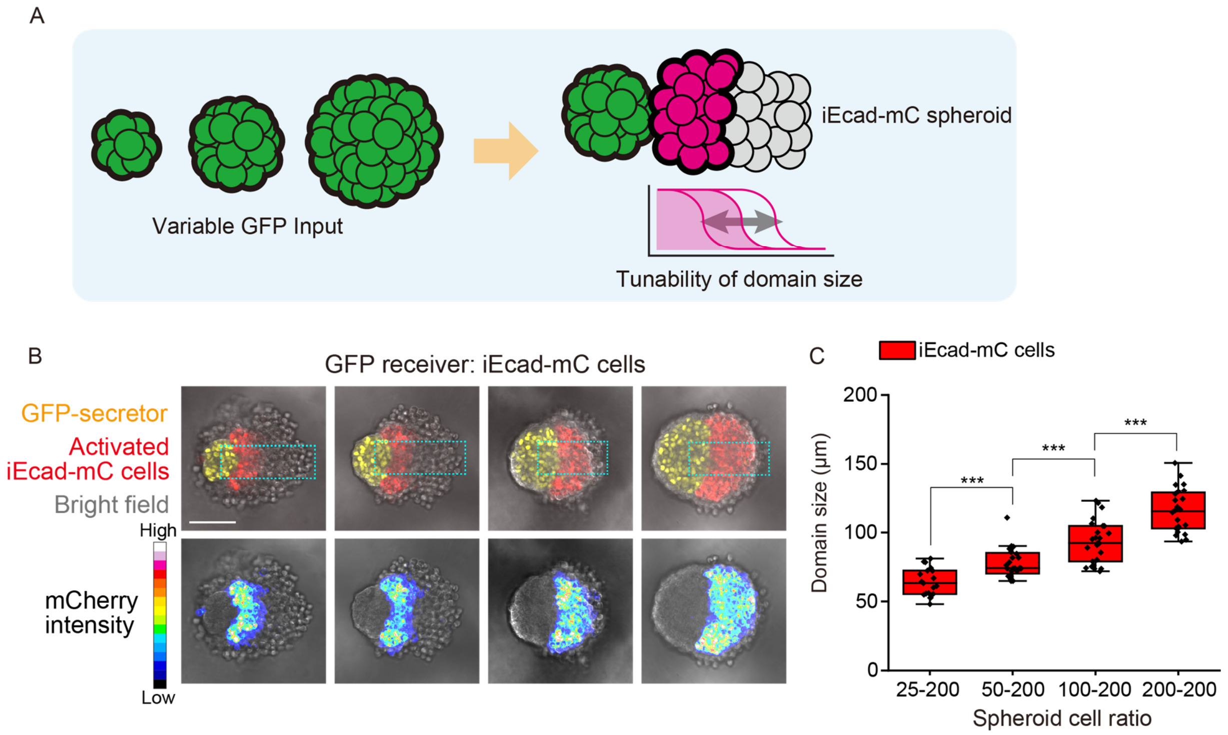

Appendix Fig. S4. Tunable tissue domain formation by morphogen-induced cadherin.

(A) Scheme of the co-culture experiments with variable sizes of GFP-secretor spheroids. **(B)** Images of synthetic tissue domains of various sizes. GFP-secretor spheroids containing 25, 50, 200, or 200 GFP-secretor cells co-cultured with spheroids composed of 200 iEcad-mC cells. mCherry distributions are visualized by 16 pseudocolors in the bottom images. Scale bar: 100 µm. **(C)** Tunability of the synthetic tissue domain size. Distances of the half intensity of mCherry in the iEcad-mC spheroids calculated from the curve fitted to the mCherry profiles in the rectangular ROIs (Appendix Fig. S4B) with the Hill equation in OriginPro software. The k values were plotted with their mean ± SD. The boxes represent the group median and interquartile range (25th–75th percentiles). The whiskers extend to the minimum and maximum data points within 1.5 times the interquartile range from the 25th and 75th percentiles, while data points beyond this range are considered outliers. Differences between two groups were determined using Welch's t-test with *** for $P < 0.001$. ($P = 9.7\times10^{-5}$ between 25-200 and 50-200. $P = 2.4\times10^{-4}$ between 50-200 and 100-200. $P = 8.6\times10^{-6}$ between 100-200 and 200-200.) 25-200 iEcad-mC: n=19, 50-200 iEcad-mC: n=23, 100-200 iEcad-mC: n=29, 200-200 iEcad-mC: n=29.

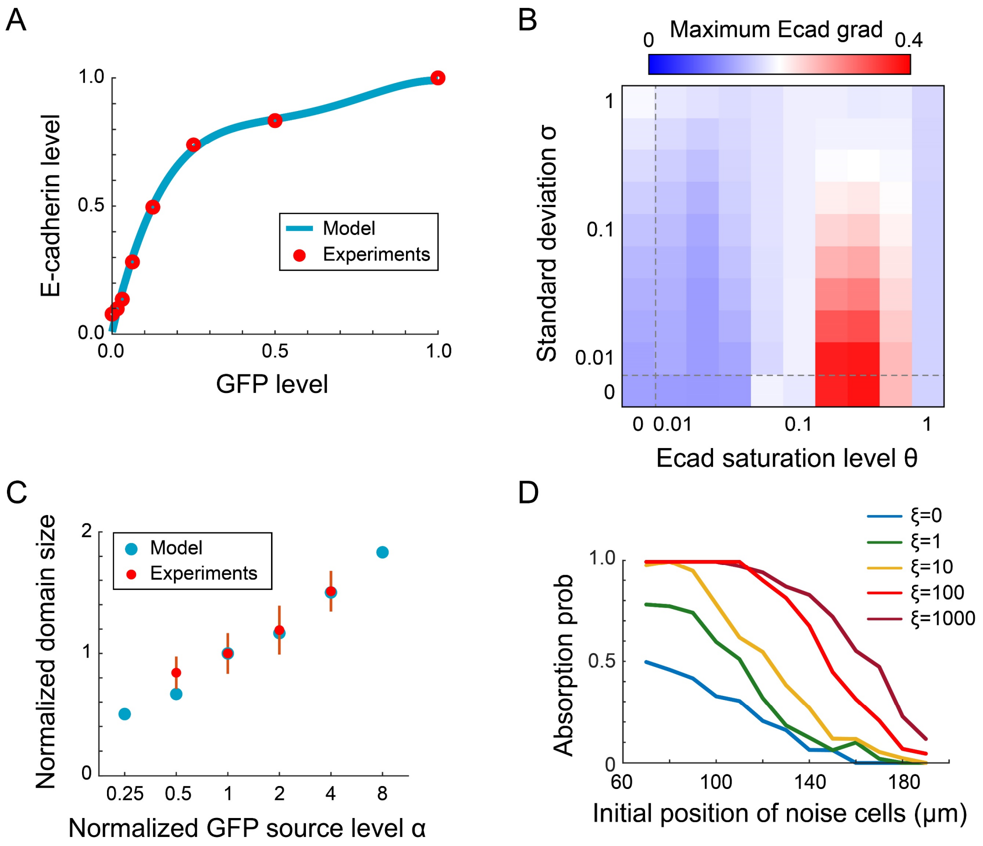

Appendix Fig. S5. Simulation analysis of the mathematical model.

(A) The correspondence curve of GFP concentration and E-cadherin-mCherry induction level in iEcad-mC cells. The red dots are plotted according to the measurement in Fig. 4C. The blue line is a fitting curve used for the simulations of the mathematical model. **(B)** The maximum gradient of E-cadherin level for the two parameters in the normal distribution of E-cadherin saturation, the mean θ and the standard deviation σ. The maximum E-cadherin gradient value tends to decrease with an increase in cell-to-cell variation σ, indicating that greater heterogeneity in the saturation level results in a less sharp boundary. However, for changes in the threshold level θ, there is an intermediate level where the E-cadherin gradient reaches its maximum. When θ is extremely high, the saturation effect is abolished, resulting in a similar outcome to the no-saturation regime. When θ is extremely low, the DAE becomes ineffective throughout the entire region, causing all cells to undergo mixing without sorting, which ultimately reduces the maximum E-cadherin gradient. n=10 for each parameter values. **(C)** Morphogen-induced cell adhesion achieves tunable tissue domain formation. The red dots are plotted according to the measurement in Appendix Fig. S4. The blue dots show the domain sizes with variable amounts of GFP source level in the mathematical modeling. n=10. **(D)** Absorption probability over the position of EACs generated with different values of ξ. Average population number at each position is 39.
