# Supplementary material for: Robust tissue pattern formation by coupling morphogen signal and cell adhesion
Source: EMBO Rep. 2024 Sep 27;25(11):4803–26. doi: 10.1038/s44319-024-00261-z (PMC11549100; doi:10.1038/s44319-024-00261-z)
Supplement: Supplementary file 15 — Expanded View Figures [file 44319_2024_261_MOESM15_ESM.pdf]

Expanded View Figures

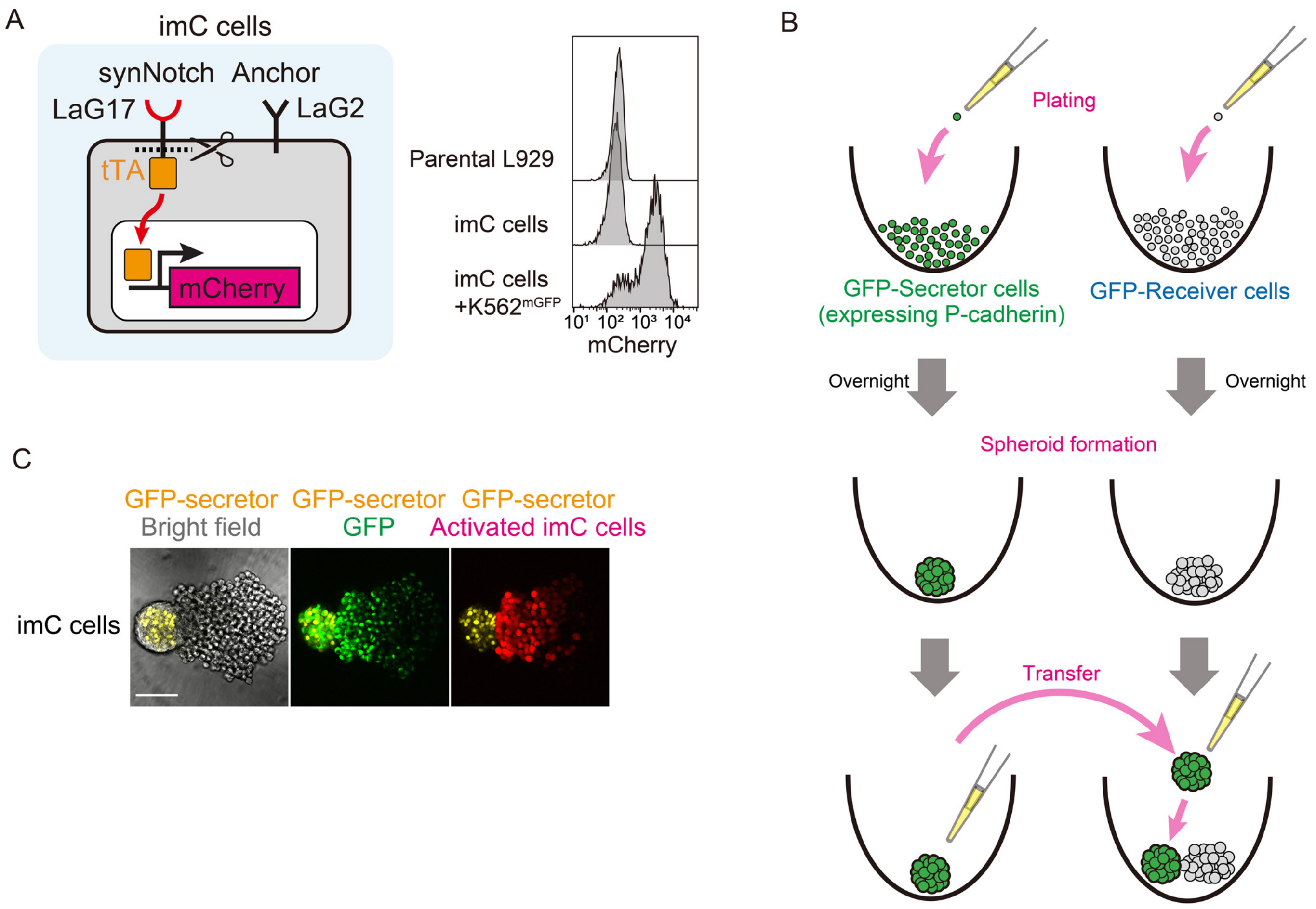

Figure EV1. Experimental setup of 3D synthetic morphogen system.

(**A**) Establishment of imC cells: imC cells express the GFP anchor protein, which captures GFP on the cell surface, and the anti-GFP synNotch receptor, which induces the mCherry reporter upon recognizing the captured GFP. The GFP-anchor protein and anti-GFP synNotch receptor utilize different nanobodies (LaG2 and LaG17, respectively) that recognize different epitopes on GFP. To confirm the induction of mCherry reporter by the anti-GFP synNotch receptor, imC cells were stimulated with K562 cells expressing membrane-tethered GFP ($K562^{mGFP}$) (Toda et al, 2018) and analyzed using flow cytometry. (**B**) Co-culture of GFP-secretor and GFP-receiver spheroids. GFP-secretor and receiver cells were separately plated and cultured overnight to form GFP-secretor and GFP-receiver spheroids, respectively. The GFP-secretor spheroid was then transferred to a well containing a GFP-receiver spheroid to initiate co-culture. (**C**) Synthetic gradient formation by GFP morphogens in imC cell spheroids. Bright-field, GFP, and mCherry channels of the synthetic gradient at 48 h. GFP-secretor cells labeled by nucleus-localized IFP2.0, which is overlaid with a yellow pseudocolor. Scale bar: 100 μm.

 © The Author(s)

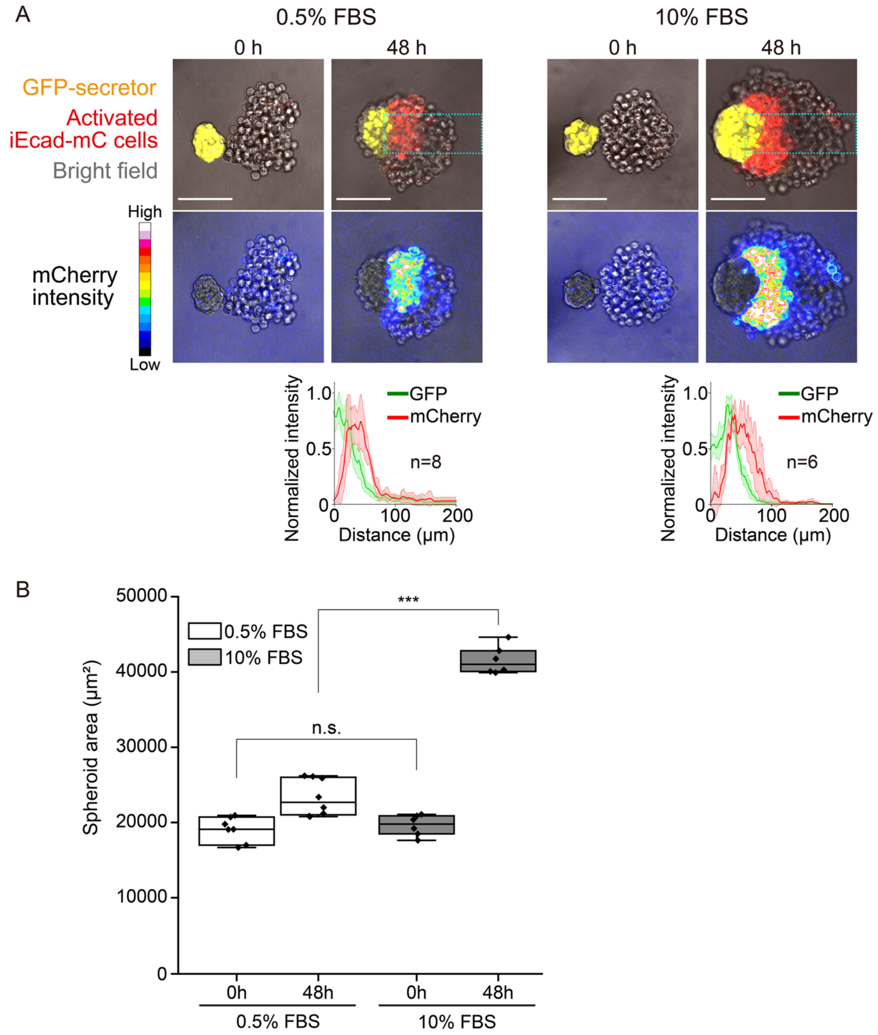

Figure EV2. Robust pattern formation in a low serum condition.

(**A**) Confocal images of iEcad-mC spheroids incubated in DMEM containing 0.5% FBS (left) or 10% FBS (right). GFP-secretor spheroids with 50 GFP-secretor cells and GFP-receiver spheroids with 200 imC or iEcad-mC cells were separately formed and co-cultured from time 0 h. Spheroid images acquired at 0 and 48 h using a confocal microscope. GFP secretor cells express nucleus-localized IFP2.0, shown as a yellow pseudocolor. Scale bar: 100 μm. mCherry distributions are visualized by 16 pseudocolors in the bottom images. Fluorescence intensity profiles within rectangular regions (green line: GFP, red line: mCherry) are overlaid at the bottom of the images. The means are indicated with bold lines (green line: GFP, red line: mCherry). Shaded areas in the graph represent ± SD. 0.5% FBS: $n = 8$, 10% FBS: $n = 6$. (**B**) The graph indicated the spheroid size at 0 and 48 h when incubated with 0.5% FBS (white box) and 10% FBS (gray box). The spheroid size slightly increased in a 0.5% FBS condition after 48 h of incubation, whereas it increased more than double in a 10% FBS concentration. The boxes represent the group median and interquartile range (25th–75th percentiles). The whiskers extend to the minimum and maximum data points. Differences between two groups were determined using Welch's t-test with *** for $P < 0.001$ and n.s. (non-significant) for $P > 0.1$ ($P = 0.51$ between 0.5% FBS at 0 h and 10% FBS at 0 h. $P = 2.2 \times 10^{-9}$ between 0.5% FBS at 48 h and 10% FBS at 48 h). The same samples were observed and measured at 0 and 48 h in each condition ($n = 8$ for 0.5% FBS, $n = 6$ for 10% FBS).

© The Author(s)

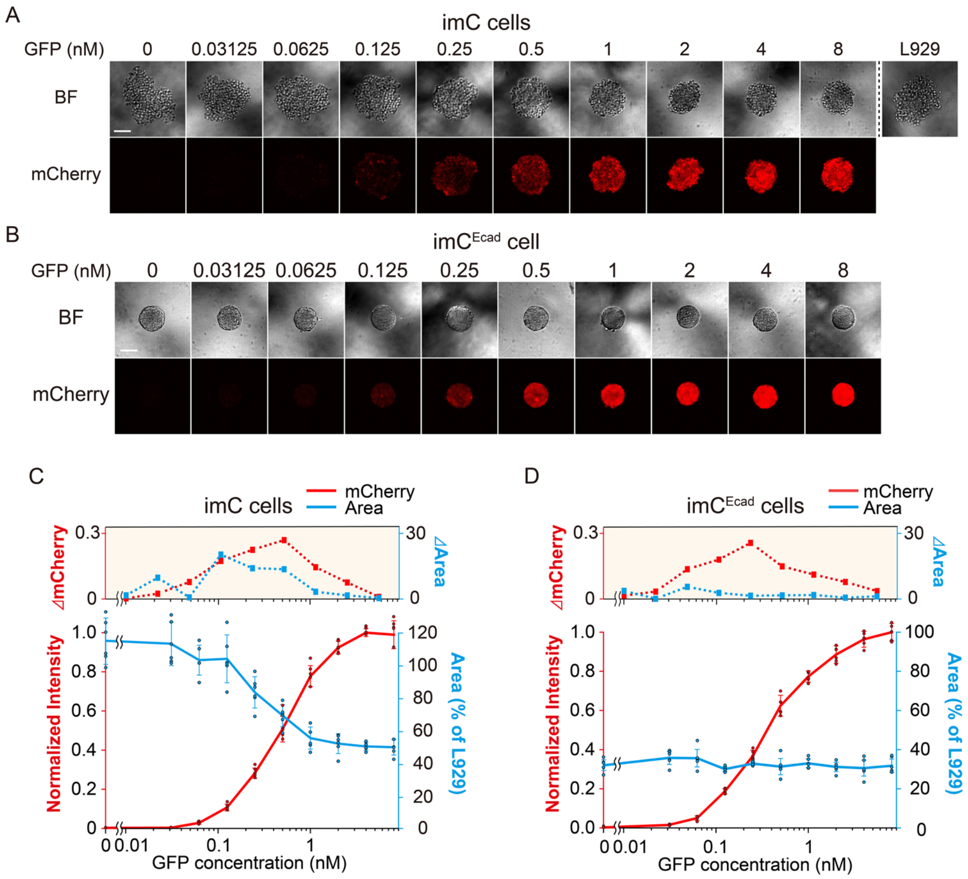

Figure EV3. mCherry induction and spheroid shape change of imC and imC^{Ecad} spheroids with variable GFP concentrations.

(**A**, **B**) Systematic analysis of the relationship between mCherry induction and spheroid shape of imC cells (**A**) and imC^{Ecad} cells (**B**). Spheroid images upon GFP concentrations ranging 0 to 8 nM. Spheroid of parental L929 is shown as control. Scale bar:100 µm. (**C**, **D**) Quantification of mCherry induction and spheroid compaction of the imC cells (**C**) and imC^{Ecad} cells (**D**). Rate of spheroid compaction quantified by comparison of the cross-sectional area of the spheroids with that of parental L929 spheroids using bright-field images. In the right graph, the left and right numbers indicate normalized fluorescence intensity of E-cadherin-mCherry and the percentage of the spheroid area relative to L929 spheroids, respectively. The difference of values between adjacent GFP concentrations is plotted in the top graph. The imC spheroids gradually shrunk in response to GFP, while the mCherry induction level was increased proportionally to the increase in GFP concentration. However, since each cell shape was round and did not form a smooth spheroid surface, this morphological change was not defined as compaction as it was caused by stronger binding to each other with higher concentration of GFP, which bridged imC cells through the trans-interaction of GFP-anchor protein/GFP/anti-GFP synNotch receptor. On the other hand, the imC^{Ecad} cell spheroids exhibited a compact morphology due to E-cadherin expression. The addition of GFP had no effect on spheroid compaction, while the induction level of mCherry reporter increased. The GFP concentrations at the curve rising (i.e., sensitivity to GFP) of imC cells and imC^{Ecad} cells were similar, indicating that E-cadherin expression does not influence anti-GFP synNotch activation. Experiments were performed in 4–6 replicates, and data are plotted mean ± SD.

 © The Author(s)

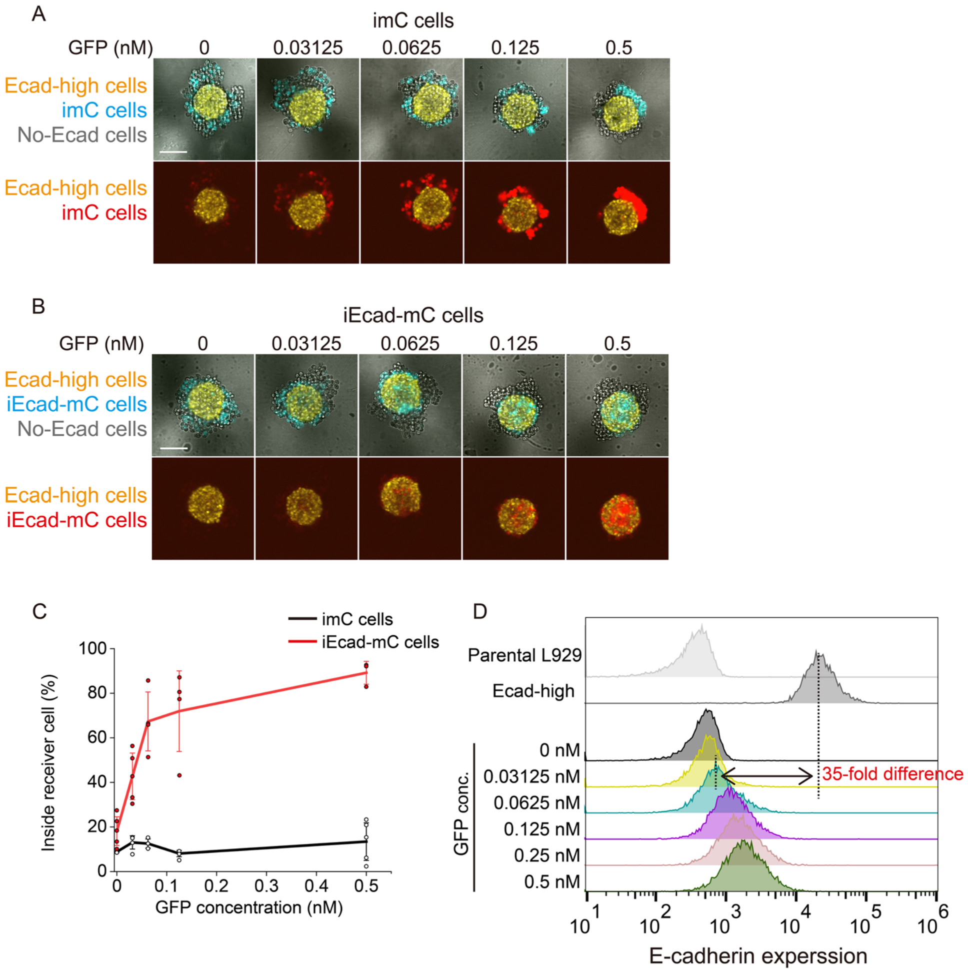

Figure EV4. Mixing of E-cadherin high-expressing cells and iEcad-mC cells.

(**A**, **B**) Activated iEcad-mC cells above a threshold level mixed well with E-cadherin high-expressing cells (Ecad-high cells). Spheroids composed of 100 Ecad-high cells were mixed with spheroids composed by combining 30 imC cells and 30 parental L929 cells (no-Ecad cells) (**A**), or 30 iEcad-mC cells and 30 no-Ecad cells (**B**) and stimulated with variable GFP concentrations. Ecad-high cells, imC/iEcad-mC cells, and mCherry/E-cadherin-mCherry are labeled with yellow, cyan, and red, respectively. Scale bar: 100 μm. (**C**) Percentage of imC or iEcad-mC cells mixed with Ecad-high cells. Results from 3–5 replicates are plotted as mean ± SD. (**D**) E-cadherin expression levels of parental L929 cells, Ecad-high cells, and iEcad-mC cells in response to varying GFP concentrations were analyzed with immunostaining using an anti-E-cadherin antibody. The mean value difference between Ecad-high and iEcad-mC cells at 0.0625 nM GFP (dashed lines) is around 35-fold.

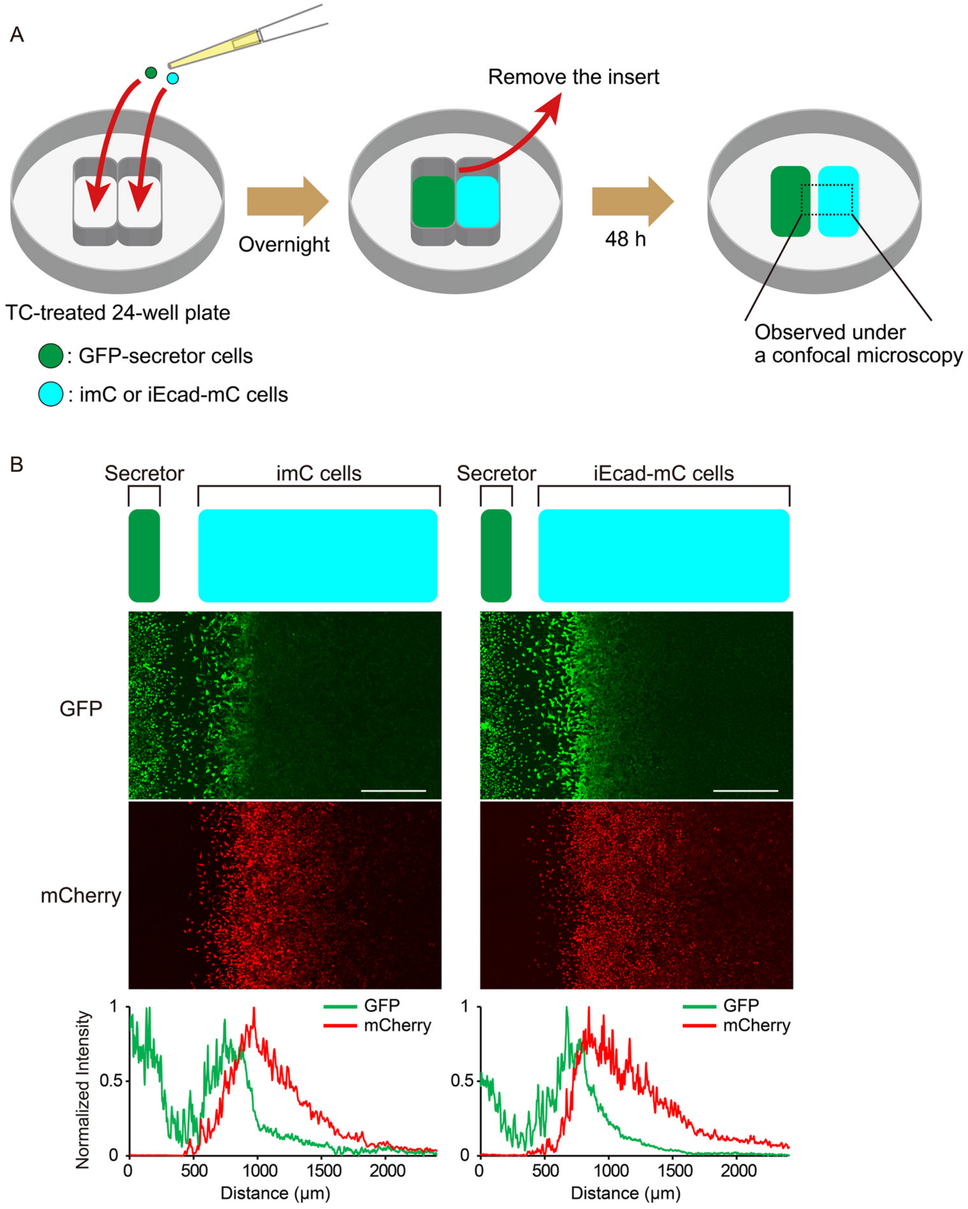

Figure EV5. Pattern formation assay in a 2D culture condition.

(**A**) Experimental setup of pattern formation assay in a 2D culture system using a two-well culture-insert. 1.6×10^4 GFP-secretor cells were plated in the left side of the insert, while 8×10^3 GFP-receiver cells were plated in the right side. The insert was removed after the cells were incubated overnight. The interaction between two regions was observed by confocal microscopy after another 48 h. (**B**) The mCherry distributions of both imC and iEcad-mC cells showed a gradient pattern. GFP-secretor cells were localized to a narrow area on the left. GFP-receiver cells were localized to the area to the right of the GFP-secretor cells, depicted in green and cyan in the upper cartoon above the confocal images, respectively. Scale bar: 500 μm. Fluorescence intensity profiles (green line: GFP, red line: mCherry) are overlaid below.

 © The Author(s)
